# Human rights approaches to suicide in prison: implications for policy, practice and research

**DOI:** 10.1186/s40352-018-0075-4

**Published:** 2018-09-14

**Authors:** Mary Rogan

**Affiliations:** School of Law, Trinity College, Dublin, Ireland

## Abstract

**Background:**

International human right standards place obligations on prison authorities to take reasonable steps to prevent suicides in prison and to investigate those which occur. Those human rights instruments contain minimum standards which states must abide by. Human rights principles can also be used in analysis of why suicides occur in prison.

**Methods:**

This paper examines human rights standards on suicide and its prevention provided by the United Nations Standard Minimum Rules for the Treatment of Prisoners, the European Prison Rules, the work of the European Committee for the Prevention of Torture and Inhuman and Degrading Treatment or Punishment, and judgments of the European Court of Human Rights. Particular consideration is given to European human rights standards in light of the literature suggesting that European approaches tend to favour the promotion of human rights. A legal research methodology is employed.

**Results:**

The paper examines key elements of human rights obligations in this field: the duty to pay particular attention to prisoners with particular mental health needs; duties to provide healthcare of an adequate standard; a duty on the prison authorities to ensure a proper information flow within prisons to identify risks; and particular duties to avoid using solitary confinement and to deploy safeguards when it is used. The paper also describes the obligations to investigate which arise after a suicide in prison: there must be an investigation instigated by the state which is independent, acts promptly and is open to public scrutiny, which is capable of giving rise to a finding of responsibility and is able to acquire relevant evidence, and which gives the next of kin of the deceased person an opportunity to participate.

**Conclusions:**

All those responsible for preventing and responding to suicides in prison must fulfil these human rights obligations, and doing so would support a culture of protecting human rights in prison. In addition, compliance with the human rights standards described here should become a factor more regularly examined in analyses of why suicides occur in prison.

## Background

The role of medical and psychological expertise in the responses to suicide and suicide prevention in prison is well established, and we are learning more about the impact of the material environment and prison regimes (Fazel et al., 2017; Sanchez et al., 2018; Stoliker, 2018). Deaths in custody also, however, involve legal obligations, and legal responses. This article examines what those obligations are, and how they might be used in examinations of the factors explaining suicide rates in prisons around the world.

Death in prison, and particularly self-inflicted death, is now a highly regulated phenomenon in many countries. In the prison system of England and Wales, for example, staff negotiate a variety of protocols and policy documents governing cell sharing, risk assessments, and first night in custody regulations (Bennett, 2016; Liebling, 1992). When a suicide occurs in prison, several investigations may be required, for example, those undertaken by the prison authorities, an inquest, an investigation by an external body such as the Prison and Probation Ombudsman in England and Wales.

Death in prison is also a focus of international human rights frameworks. The United Nations and the Council of Europe have both produced statements of principle seeking to prevent suicide in prisons and to promote investigation of them. These principles are found in international legal instruments, but also in the work of international bodies responsible for the monitoring of prisons. At the European level, the European Court of Human Rights has also considered deaths occurring in prisons in Council of Europe member state countries. Within Europe in particular, human rights principles are considered to have influenced its prison systems for the better, particularly concerning material conditions and the promotion of rehabilitation as a goal of sentences (Snacken, 2010; Van Zyl Smit & Snacken, 2009). While the empirical basis for such claims may be somewhat limited, the promise of human rights frameworks is that, through compliance with them, a more just system which promotes human dignity will result.

This article presents a human rights-based approach to suicide in prison, focusing on its prevention and the importance of investigations when suicides take place. It is intended to provide an overview of the human rights obligations on prison staff and governments in preventing and responding to suicides in prison which act as a guide to minimum standards for prison and healthcare managers dealing with this area. These principles can and should be used by those authorities to review their practice on a regular basis. In addition, the paper argues that human rights compliance should be used directly as a factor in studies of why suicides occur in prisons. Many empirical studies of the risk factors for suicides in prison examine indicators such as overcrowding, the general suicide rate in the population and so on. A sensible hypothesis, it is submitted, is that prisons which do not fulfil their obligations under human rights law to take steps to prevent suicides and investigate them when they occur, will see higher rates of suicide. A first step towards such measurement might be to use the human rights principles articulated in this piece as indicators of human rights compliance and then include them in studies examining the factors associated with prison suicides at the prison and country level.

## Methods: A legal research methodology

This paper seeks to describe and analyse human rights principles governing suicide in prison. The purpose of describing those principles is to provide a clear summary of the norms and standards governing this subject, found in a wide variety of legal sources. The analysis section then seeks to categorise the various principles arising into clusters, examined under the subheadings used below.

The sources forming the basis for this description are as follows. First, the paper examines the United Nations Standard Minimum Rules for the Treatment of Prisoners. It also takes three European sources: the European Prison Rules; standards produced by the Council of Europe’s Committee for the Prevention of Torture and Inhuman and Degrading Treatment or Punishment; and decisions of the European Court of Human Rights. Other human rights instruments govern this field. The United Nations Standard Minimum Rules for the Treatment of Prisoners and the European Prison Rules are international standards, the former having a global reach, while the latter extends to all states which are parties to the Council of Europe (presently 47 countries). These standards reflect global and European perspectives on what a minimum standard of treatment for prisoners should be and a distillation of international perspectives and experiences regarding treatment in prison. One might say they reflect something of a collective wisdom concerning a basic standard for penal conditions and the content of penal policies and practices. The European Prison Rules, dating from 2006, are not binding on states, and there is no enforcement mechanism by which they can be imposed on prison systems. However, the European Court of Human Rights refers to them regularly in its analysis of whether there has been a breach of the European Convention on Human Rights in a particular case (Van Zyl Smit & Snacken, 2009). The United Nations Standard Minimum Rules were adopted in 2015 following a long process of discussion and consultation, and a considerable role for experts (Rodley, 2015). They are known as the Mandela Rules having been concluded in South Africa. Between these two sources, we find what at least purports to be a broad international consensus about the human rights standards which should apply in the prison context. It is for this reason that these two sources have been selected.

The European Committee for the Prevention of Torture and Inhuman and Degrading Treatment or Punishment (CPT) was established by means of a Convention concluded in 1989 (Bicknell & Evans, 2017). The CPT is empowered to visit and report on places where people are deprived of their liberty, such as prisons, police stations, immigration detention centres, psychiatric institutions and social care homes in all 47 member states. Its members, which are nominated by the governments of member states, comprise experts in law, medicine and penal administration. It has extensive powers conferred by its originating Convention to enter places of detention and to speak in confidence to prisoners, staff and others. It does not have powers of enforcement, but relies on publication of its reports and a moral standing to promote the adoption of its recommendations. Since its inception, the CPT has carried out hundreds of country visits and made recommendations across many aspects of penal policy and practice, including on the question of the prevention and investigation of suicides in prison. The CPT is considered to have had a significant influence on penal practice within Europe, and also on the decisions of the European Court of Human Rights. As Daems and Robert state: “the CPT, was, at the time it became operational …, a unique institution and it has, in many ways, been perceived as an example and forerunner for similar institutions across the globe” (Daems & Robert, 2017, p. 3). The CPT has created a series of standards which guide its approach to visits and which provide a blueprint for prison systems seeking to prevent torture and inhuman and degrading treatment. It is included in this study because it is recognised across Europe as an authoritative standard-setter for human right protection in prisons.

The European Court of Human Rights is a judicial body which has addressed the question of suicide in prison on a number of occasions. The European Convention on Human Rights contains the right to life in Article 2, and the right to freedom from torture and inhuman and degrading treatment in Article 3. If it decides that there has been a breach of the Convention, it will make a declaration to that effect and can award compensation to the victim of the breach. The manner in which a declaration is dealt with depends on domestic law. This source has been chosen because the European Court of Human Rights provides authoriative determinations of matters concerning the interpretation and application of the European Convention on Human Rights, and because it can hold states to account for violations of that Convention.

To fulfil the first aim of the study, i.e. to describe the relevant legal principles, a legal research methodology was employed. First, following the selection of the Mandela Rules and European Prison Rules, both sources were searched for the terms “death”, “suicide”, “die”, and “self-inflicted”. Both sources were also read in their entirety to ensure that no relevant provision was missed. This analysis was completed during the month of March 2018. In order to search for relevant standards and observations provided by the CPT, the HUDOC database for reports of the CPT was also searched. This database contains all reports created by the CPT following its country visits, as well as its General Reports produced annually and thematic reports. This database using the terms “suicide” and “self-inflicted death”. This database was searched on April 30 and May 1 2018. Because the General Reports of the CPT contain principles of general application, rather than directed to a particular state and set of circumstances, these were examined first. Reports from visits to states for the prior five years generated by this search were then examined. A specific search was also made of CPT standards and reports on the topics of solitary confinement. This was chosen because of the known effects of solitary confinement and risks of self-harm and suicide. The view of the CPT on this subject was therefore important.

A search was aso undertaken for relevant cases dealing with instances of suicide in prisons. Ths involved accessing the European Court of Human Rights’ HUDOC database and entering in the following search terms: “suicide AND prison”; “Article 2 AND prison”; “death in custody”; “self-inflicted death AND prison”, as well as using the search term “suicide AND prison” in the search mechanism examining Articles 2 and Article 3 specifically. This database was searched on April 23 and 24 2018. The searches of the HUDOC database generate results by date and relevance. It should be stated that some of the cases are well known as having laid down important statements of principle, and are recognised as such in legal analyses of the subject (Livingstone et al., 2015; Rogan 2014; Van Zyl Smit and Snacken, 2009). Cases which are more recent than those decisions were therefore explored to assess whether they contained any new statements of principle.

Legal research is notable for its tendency not to explain its research methods (McCrudden, 2006) and legal research methods do not follow precisely those of other disciplines. When assessing a body of caselaw and legislation, legal researchers do not, generally, take note of the number of relevant cases but rather identify those with the most precedential value, usually because of the status of the court involved, and then examine how later decisions build on and apply those principles. Cases which simply apply the principles and give no further guidance of general application were not described in the analysis below. The type of analysis familiar to those engaged in systematic literature reviews or meta-analyses for example, is not involved, with legal researchers instead following a chain of precedent. This is not to say that legal research methodology is not rigorous, indeed any person making arguments in court will soon find out if their reasoning is flawed, but it does not lend itself to an orderly descrption of the precise process. Perhaps the most straightfoward way to conceive of this method, which can be made unduly mystical by legal researchers, is to think of it as the researcher organising and synthesising caselaw and other legal sources into coherent patterns and categories, using legal training to understand the relevant hierarchies of those sources (Hutchinson and Duncan, 2015).

A word must also be said on why three European sources have been included and only one global source. The inclusion of the European sources is based on literature to the effect that the European way in punishment is distinctive and one which has promoted the protection of human rights as well as rehabilitation as a goal of penal policy (Daems & Robert, 2017; Snacken & Dumortier, 2012; Van Zyl Smit & Snacken, 2009). This scholarship suggests that European human rights institutions have done much to uphold human rights in Europe’s prisons, something often presented in contrast to a more punitive United States (Girling, 2006; Simon, 2014; Whitman, 2003). While there are many reasons to be critical of European prisons, the approach evident in its human rights instruments is considered in this literature to be distinctive, and valuable. As such, focusing on these sources, it is submitted, provides useful guidance for states seeking to promote a human rights-compliant approach concerning suicide in prison.

## Human rights standards and suicides in prisons

The human rights standards which apply in this area fall into two main categories. The first concerns the duty to take reasonable steps to prevent suicide, while the second concerns the duty to investigate incidents when they do occur. These obligations are derived directly from the caselaw under the European Convention on Human Rights, as described below. These are enforceable legal obligations. Similar duties are placed on states which sign up to the European Prison Rules and Mandela Rules, but these are not directly enforceable. Within the first obligation, a review of the legal principles shows that four key areas arise. First, there must be special attention given to prisoners with particular mental health needs; second duties are placed on medical professionals, but are also placed on prison authorities to provide healthcare and support those professionals; thirdly a proper information flow within prisons has been identified as important by these principles; finally, the particular context of solitary confinement has also been given attention. The next section of the paper presents the findings of this distillation of the evidence derived from these legal sources.

### The duty to take reasonable steps to prevent suicides in prisons

Under Article 2 of the European Convention on Human Rights, prisoners have the right to life and penal authorities are under a duty not to take life intentionally or by a disproportionate use of force in the pursuance of the legitimate aims mentioned in Article 2 itself (in defence of any person from unlawful violence, in order to effect a lawful arrest or to prevent the escape of a person lawfully detained, or in action lawfully taken for the purpose of quelling a riot or insurrection). Beyond this, these authorities must also take steps in order to prevent deaths where possible. This obligation is particularly acute in the case of those who are in the care and custody of the state in light of the inherent vulnerability of that position.

This preventive obligation applies in circumstances where the threat to life arises from the prison, from prison staff, from another person in prison, or from the person themselves. If the authorities did not do all they could be reasonably expected to do to prevent a person taking his or her life, then they will have breached Article 2. These principles have been elaborated upon in a series of cases. In *Keenan v. United Kingdom* (5 April 2001 33 EHRR 38), for example, a prisoner with mental illness took his own life in circumstances where he had been placed in segregation for seven days for assaulting two prison officers, and had his sentence extended by a further 28 days. The deceased’s mother argued that the UK government had failed to vindicate her son’s right under Article 2 in failing to prevent his suicide. The Court recognised the principle that prison authorities are obliged to safeguard prisoners’ lives. However, in the particular circumstances no violation of Article 2 was found. While the risk of the prisoner’s suicide was known, the authorities had taken reasonable action in the circumstances of his behaviour prior to his death, which did not foreshadow what was to transpire. The Court stated the test for a breach of Article 2 in such circumstances was whether the prisoner was at an immediate risk of suicide and, if so, whether the authorities did all they could be reasonably expected to do. However, a breach of Article 3 was made out. The lack of medical notes and monitoring of the prisoner along with the imposition of segregation was considered to be incompatible with the proper treatment of a person with mental illness.

The seminal case of *Edwards v. United Kingdom* (application no. 46477/99, 14 March 2002) held that where the prison authorities know or ought to know of a threat to life and do not act, they will not have fulfilled their obligation under Article 2. in the *Edwards* case*,* a threat came from a person sharing a cell which should have been picked up during screening on that person’s arrival into the prison. This reasoning applies to all deaths in custody. As the case of *Kats v Ukraine* (application no. 29971/04, 18 December 2008) has held, the very fact of dying in custody in suspicious circumstances raises a concern that Article 2 has not been complied with, and the right to life has not been vindicated. The Court noted that Article 2 imposes an obligation not only to refrain from causing death intentionally or by disproportionate use of force, but also to take any necessary measures to secure the protection of the lives of individuals.

In *Coselav v. Turkey* (application no. 1413/07, 9 October 2012) a sixteen-year-old prisoner took his life by hanging from the iron bars of his prison cell using bed sheets. The child had previously made two attempts at suicide. Following one such incident he had been disciplined. He also sent numerous letters to the prison authorities requesting to meet the Governor urgently to discuss his personal problems. He had requested a transfer to another prison, but this was refused. After hearing this, he engaged in destructive behaviour. On the day of his death, he had been taken to the prison hospital after repeatedly hitting his head against the wall of his cell. He was returned to his cell following treatment. In the view of the Court, the prisoner was at clear risk of suicide and his psychological problems had been well documented. Despite their awareness of the risk of suicide, the authorities had not taken the necessary precautions to prevent his death, particularly by bringing him back to his cell following the incident in which he had hit his head off the wall of his cell. This, combined with the failure to provide him with any medical or other specialist care, was a violation of Article 2.

It is clear that where the authorities know of a risk of suicide they must take reasonable steps to prevent that suicide, and document these steps. Reasonable does not mean, however, that the person’s autonomy or dignity should be wholly negated in order to prevent the death. While the duty to take all reasonable steps to prevent a suicide is well established, the question of what constitutes reasonable steps is a difficult one. Advice from a doctor or other health-care professional which is not followed would clearly raise substantial concerns that reasonable steps were not taken. Having no response, or a response which does not conform with generally accepted medical practice to concerning behaviour would raise similar problems for the prison authorities. Having no screening, or inadequate health-care screening on arrival would also be likely to fall within the meaning of unreasonable. Some additional guidance on how prison authorities ought to act can be gleaned from the reports of the CPT, the Mandela Rules and the European Prison Rules. These principles are discussed below.

### Prisoners with mental disabilities and/or health conditions

As noted above, the European Court of Human Rights has found the improper treatment of a person with mental illness when that person took his own life amounted to inhuman and degrading treatment. Rule 12.1 of the European Prison Rules states clearly that people suffering from mental illness and whose state of mental health is incompatible with detention in a prison should be detained in an establishment specially designed for that purpose. The rules recognise, however, that such people are detained in prisons, and exhorts the authorities to put in place special regulations to take account of their status and needs (Rule 12.2). The Mandela Rules make it clear that a person who is diagnosed with severe mental disabilities and/or health conditions for whom staying in prison would mean an exacerbation of their condition must not be detained in prisons and should be transferred to mental health facilities as soon as possible. Other prisoners with mental disabilities and/or health conditions are permitted to be observed and treated in specialised facilities under the supervision of qualified health-care professionals. Psychiatric treatment shall be provided for all who need it (Rule 109).

The importance of proper screening at admission, an examination by medical staff and keeping of records of visible injuries and complaints about prior ill-treatment is also evident in the European Prison Rules. Rule 16 of the European Prison Rules requires that prisoners receive a medical examination as soon as possible after admission. The CPT has also emphasised the importance of medical screening on arrival to a prison, and highlighted the role of the reception process in suicide prevention (Third General Report and 26th General Report). The need for screening to ascertain if a prisoner is at risk of suicide has been mentioned by the CPT on several occasions (e.g. Bulgaria, 2017; United Kingdom Sovereign Base Areas on Cyprus, 2017; Lithuania, 2016; Liechtenstein, 2016; Malta, 2015; Kosovo*, 2015; Sweden, 2015).

### The role of medical professionals and other staff

A situation where prison authorities do not provide adequate healthcare for prisoners and this can be linked to the death of the person is likely to be considered to fall outside a definition of reasonable steps. The European Prison Rules require medical services in prison to seek to detect and treat physical or mental illnesses or defects (Rule 40.4) and that all necessary medical, surgical and psychiatric services including those available in the community shall be provided (Rule 40.5). These rules also require each prison to have personnel suitably trained in health care (Rule 41.4). A medical practitioner or qualified nurse must pay particular attention to diagnosing physical or mental illness (Rule 42.3.b), dealing with withdrawal symptoms resulting from the use of drugs, medication or alcohol (Rule 42.3.d), and identifying any psychological or other stress brought on by the fact of deprivation of liberty (Rule 42.3.e). Medical practitioners shall report to the prison director [governor] whenever it is considered that a prisoner’s physical or mental health is being put seriously at risk by continued imprisonment or by any condition of imprisonment, including conditions of solitary confinement (Rule 43.3).

There is considerable emphasis on the role of health-care professionals in the Mandela Rules. Rule 30 of the Mandela Rules states that a physician or other qualified health-care professionals shall talk with an examine every prisoner as soon as possible following his or her admission and thereafter as necessary. The rules require that particular attention be paid to identifying health-care needs, any ill-treatment prior to admission, as well as any signs of psychological or other stress brought on by the fact of imprisonment, including, but not limited to, the risk of suicide or self-harm. All appropriate individualised measures or treatment should then be taken. There is a duty on the physician to report to the prison director whenever he or she considers that a prisoner’s physical or mental health has been or will be injuriously affected by continued imprisonment or by any condition of imprisonment (Rule 33). Under Rule 46, health-care personnel shall report to the prison director, without delay, any adverse effect of disciplinary sanctions or other restrictive measures on the physical or mental health of a prisoner and advise the director if they consider it necessary to terminate or alter those measures for physical or mental health reasons (46.2). Rule 46.3 gives health-care personnel the authority to review and recommend changes to the involuntary separation of a prisoner in order to ensure that such separation does not exacerbate the medical condition or mental or physical disability of a prisoner. Rule 31, furthermore, requires that all prisoners who complaint of physical or mental health issues or injury should have daily access to a physician or other qualified health-care professional (Rule 31). The CPT has also noted the importance of good practices for the distribution of medication and supervision of prescribed dosages (United Kingdom Sovereign Base Areas on Cyprus, 2017).

The need for proper access to medical services which are comparable to those available in the community is also a feature of human rights instruments. The European Prison Rules, in rule 40, exhort prison authorities to provide all necessary medical, surgical and psychiatric services, including those available in the community to a person in prison. Rule 47.1 states that specialised prisons or sections under medical control shall be available for the observation and treatment of prisoners suffering from mental disorder or abnormality, while rule 47.2 states that the prison medical service shall provide for the psychiatric treatment of all prisoners and pay special attention to suicide prevention. Under rule 46, people who need treatment which is not available in prison should be transferred to outside hospitals. These principles are also reflected in the Mandela Rules, which also promote the need for interdisciplinary teams acting with full clinical independence in the prison setting (rule 25).

The position of medical staff in prison can be a rather precarious one, and human rights instruments oblige states to protect them. Medical staff have crucial roles to play in preventing deaths, but also in documenting incidents. The CPT has described medical professionals in prisons as “potentially staff at risk” (Third General Report) as they may come under pressure from security staff to amend their advice on the grounds of operational considerations, or, to participate in, or justify acts of torture, inhuman or degrading treatment. These protections are also given considerable attention in the Mandela Rules, with rule 27 stating that clinical decisions may only be taken by the responsible health-care professionals and may not be overruled or ignored by non-medical prison staff. This is a critical recommendation in the context of supporting the prevention of death in prison, but one which is too-often in tension with operational priorities.

The CPT further states that the prevention of suicide must not be confined to prison healthcare staff, but awareness-raising must take place throughout the prison. Prison staff must be trained in recognising indicators of suicidal risk (Third General Report; Serbia, 2017) and monitoring (Greece, 2015). Rule 81.3 of the European Prison Rules states that staff who work with mentally ill prisoners should receive specific training for their specialised work. More generally, the CPT has stated strongly that without a manageable prison population size, safety in prisons which are under the control of staff, suicide prevention mechanisms will be unworkable (United Kingdom, 2016). The broader context of the scale, nature and approach to imprisonment is intimately connected with suicide prevention and response.

### Record keeping and oversight

Proper record keeping and the transfer of information are both critical areas for the prevention of suicide in prison and in supporting robust investigations of them. This is recognised in the international human rights standards. Rule 15 of the European Prison Rules state that, at admission, a record should be kept of any visible injuries and complaints about prior ill-treatment (Rule 15). The CPT has stated that a medical file should be compiled for each patient and be transferred with the person. It has also emphasised the need for a proper flow of information within an establishment and between establishments (and their health care services) about people who have been identified as being potentially at risk (Third General Report). The commentary to the European Prison Rules describes record keeping as a vital protective measure, with the Mandela Rules going further, requiring states to prevent unauthorised access or modification of prison records. The Mandela Rules require a secure audit trail to be kept (Rule 6). The CPT has also recommended the creation of a central register to record all incidents of suicide in a prison to allow management and external monitors to gain a clear picture of the situation in the prison (Cyprus, 2017), and has noted the importance of ensuring a proper flow of information within a prison (Malta, 2015). It has also advocated for clear criteria on how deaths are classified as suicides (The Former Yugoslav Republic of Macedonia 2016).

### Solitary confinement

The harms of solitary confinement, especially for those with mental disorders are now well known. Within human rights law the clearest statement of the harms of solitary confinement and the need for it to be limited within human rights standards comes in the Mandela Rules. Rule 44 of those rules provides a definition of solitary confinement, being confinement for 22 h or more a day without meaningful human contact. Prolonged solitary confinement refers to solitary confinement for a time period in excess of 15 consecutive days. The Mandela Rules state that solitary confinement “shall be used only in exceptional cases as a last resort, for as short a time as possible and subject to independent review” (Rule 45). Furthermore, it can only be imposed by a competent authority and should not be an automatic consequence of a particular sentence. The rules specifically prohibit the imposition of solitary confinement in circumstances where a prisoner has mental or physical disabilities when their conditions would be exacerbated by such measures (Rule 45.2). Other standards which prohibit the use of solitary confinement for women and children are reiterated in the Mandela Rules. Indefinite and prolonged solitary confinement are prohibited under the rules, as is placement of a prisoner in a dark or constantly lit cell (Rule 43).

The CPT has also stated that solitary confinement can, in certain circumstances, amount to inhuman and degrading treatment, and all forms of it should be as short as possible (CPT Second General Report). In its 21st General Report of 2011, the CPT described the effects of solitary confinement as potentially “extremely damaging” (at para. 53). It further noted that the importance of positive doctor-patient relationships means that medical personnel should never participate in any part of a decision-making process resulting in any type of solitary confinement, unless the measure is to be applied for medical reasons (at para. 62). The CPT has also stated its view that the use of segregation for an inmate at serious risk of attempting self-harm or suicide is totally unsuitable and unacceptable. Instead, such individuals should be placed in a closed hospital environment with suitable equipment and staff (United Kingdom, 2016). The CPT has also called for access to purposeful activity to counteract the negative effects of segregation (Finland, 2014).

### The duty to investigate suicides in prison

The second main strand within human rights frameworks as they apply to the question of suicide in prison concerns their investigation. Article 2 of the European Convention on Human Rights has been interpreted as placing a duty on a state to ensure that deaths in custody are investigated. The positive obligation to protect life implies a duty to investigate these deaths. As the European Court of Human Rights has declared in *Kats v. Ukraine*:

Article 2 entails a duty for the State to ensure, by all means at its disposal, an adequate response – judicial or otherwise – so that the legislative and administrative framework set up to protect the right to life is properly implemented and any breaches of that right are repressed and punished (at para 115).

The principles governing this area are largely derived from decisions concerning the use of lethal force by the police. Based on this jurisprudence, the case of *Edwards v. United Kingdom* laid down the principles which must be followed when investigating a death in custody. An investigation must be:

- Instigated by the state (a negligence action taken by the deceased’s family will not suffice);
- Independent of those implicated in the death, both institutionally and in practice;
- Prompt and be open to public scrutiny
- Be capable to giving rise to a finding of responsibility and to enable the eventual prosecution of those responsible through the acquisition of relevant evidence;
- Give the next of kin of the deceased an opportunity to participate.

The European Court of Human Rights has not laid down specific guidance on the precise type of investigation which will comply with Article 2, but the Court has examined the compliance of several kinds of enquiry with Article 2. In *Edwards* a non-statutory inquiry was commissioned by the Prison Service, the County Council in which the prison was located, and the area’s local health authority. It could not compel witnesses, including two prison officers on duty on the night in question. Another critical problem related to the degree of participation of the Edwards family. The parents of Mr. Edwards were only allowed to attend the inquiry when giving their own evidence and could not receive the evidence of others until the final report was published. They could not put questions to any witnesses and were not legally represented. These two flaws meant that the enquiry did not comply with Article 2.

Concerning independence, the Court has held that it must be both institutionally and practically independent. It must also be capable to giving rise to a finding of who was responsible and holding them to account. This means the investigation must be able to ascertain the circumstances in which the death took place as well as any regulatory shortcomings, along with identifying any state officials or bodies which were involved (*Kats v. Ukraine)*. This further implies that the authorities must take reasonable steps to secure evidence about the incident, such as eyewitness reports, forensic evidence and the result of autopsies.

Having a prompt investigation is considered by the Court to be essential in maintaining public confidence in the authorities’ adherence to the rule of law and in preventing any appearance of collusion in or tolerance of unlawful acts (*Edwards v. United Kingdom*). It also considered that the passage of time can affect the amount and quality of evidence, cast doubt on the good faith of the investigators and prolong the ordeal for the family involved. There is, however, no specific guidance on what precise amount of time is considered to be too long. A time frame of three and a half years between the death and the report was acceptable in *Edwards* for example, but delays in the order of decades will not be tolerated.

Public scrutiny is another essential feature of an Article 2-compliant investigation. There must be a “sufficient element of public scrutiny of the investigation or its results to secure accountability in practice as well as in theory” (*Edwards v. United Kingdom,* at para 73; see also *Shevchenko v. Ukraine* no. 32478/02, 65, April 4 2006). This may be fulfilled by the publication of a report into an investigation once that meant that the relevant state agents were rendered practically as well as theoretically accountable for their actions. However, where there are particularly horrendous circumstances, features of vulnerability on the part of the deceased prisoner, or particular failures by the authorities, a full public inquiry might be necessary (*Edwards v. United Kingdom*).

In all cases, the family must be involved in the inquiry to the extent necessary to safeguard their interests. At a minimum, the family of a deceased prisoner must be involved in any inquiry into his or her death. In *Kats v. Ukraine* the effective exclusion of the family of a prisoner who had died from medical complications in custody from the subsequent inquiry was at issue. The lack of even basic information about its progress was considered to breach the requirement that the interests of the next-of-kin be safeguarded as well as the need for public scrutiny.

In *Coselav v. Turkey* (application no. 1413/07, 9 October 9 2012) the parents of a prisoner who took his own life were informed of his death 13 days after it occurred, preventing them from taking part in the crucial early stages of the investigation. During a criminal investigation there had also been no attempt to enquire as to the reasons for the prisoner’s suicide and the possible responsibility of the authorities. A claim taken by the family seeking compensation could not remedy these failures. As a result, Article 2 was violated.

The European Prison Rules, by contrast, contain comparatively limited discussion of how deaths in custody should be investigated. Rule 24.9 states that when a prisoner dies, the authorities must immediately (unless the prisoner has required them not do) inform his or her spouse or partner, or nearest relative or any other person previously designated by the prisoner. The CPT, however, has drawn attention to concerns about the investigation of deaths in prison on several occasions. For example, during its visit to Portugal in 2016, the CPT recommended that a thorough investigation be carried out into every death in prison by an independent authority to ascertain, inter alia, the cause of death, the facts leading up to the death, including any contributing factors and whether the death might have been prevented. An analysis of each death to consider what general lessons may be learned for the prison and whether any systemic, nationwide measures are needed was also advocated (Portugal, 2016). The CPT has also suggested that Austria should extend the requirement to notify of the Office of the Ombudsman Board of every death, suicide and suicide attempt in police detention to prisons (Austria, 2014).

The Mandela Rules also provide for the investigation of a death in prison. Rule 71 explicitly requires an independent investigation into any custodial death which is mandated to conduct prompt, impartial and effective investigations into the circumstances and causes of the death. The prison administration must cooperate fully with the authorities and ensure all evidence is preserved. The rules go further than the European Prison Rules in another respect when, in rule 72, they say that “the prison administration shall treat the body of a deceased prisoner with respect and dignity” and returned to the next of kin as soon as possible. A checklist for assessing compliance with the Mandela Rules notes that custodial deaths should be subject to a standardised process of scrutiny and an external investigation (United Nations Office of Drugs and Crime, 2017).

## Discussion

This review of human rights frameworks indicates that the following points of guidance for prison authorities and states wishing to ensure a human rights-based approach to the issue of suicide in prison must be considered and respected. These can be thought of as minimum standards which should inform how prison managers and staff deal with this issue.

1. The prison authorities must take all reasonable steps to prevent suicides;
2. What constitutes ‘reasonable’ will vary depending on the circumstances, however, at a minimum, prison authorities must ensure:
  - Those suffering from mental illness whose illness is incompatible with detention in prison, or which is exacerbated by detention, should be transferred to mental health-care settings;
  - Those with mental illnesses short of this standard must be observed and treated in specialised facilities involving qualified health-care professionals;
  - There is a screening programme in place as soon as possible after admission which examines suicide risk factors;
  - There are health-care services and professional staff in prison charged with detecting and treating mental illness;
  - All necessary health-care services, including those available in the community, should be provided to a prisoner;
  - Clinical decisions are respected and are not ignored or overruled by prison staff.
  - Prisons have a suicide prevention strategy.
3. There must be adequate and properly maintained systems of record-keeping, including a proper information flow concerning prisoners at risk.
4. Solitary confinement should not be used for those with mental disorders, used only in exceptional circumstances in other cases and for as short a time as possible; the use of segregation for prisoners at serious risk of attempting suicide is prohibited;
5. When a suicide does occur in prison, there must be an independent investigation into it. That investigation must be instigated by the state, act promptly, be open to public scrutiny, be capable of giving rise to a finding of responsibility and give the next of kin an opportunity to participate.

In addition, the guidance and the human rights standards distilled and described here, also provide a basis for scholars to add another dimension into studies of the factors influencing suicide rates in prison. It is notable that such studies have examined what might be considered proxies of human rights compliance (such as overcrowding levels and contact with families), but have yet to explicitly include human rights compliance rates in this analysis (Fazel et al., 2017; Stoliker, 2018). It is submitted that another indicator to be used in these kinds of models should be the human rights compliance of the relevant prison system in the specific areas of the prevention and investigation of suicides. How could this be done in practice? The first step is to define the criteria by which a judgment is made as to whether the prison system is compliant with human rights standards or not. This article has provided criteria which could be used e.g. whether there is adequate record keeping and a flow of information concerning a prisoner at risk. The second task is to determine whether those criteria are met or not. This kind of measurement would be challenging. The sources for making such a judgment would include the reports of domestic and international inspection and monitoring bodies such as the CPT, reports of local non-governmental organisations and domestic court cases. Using those sources, the researcher could ascribe a score for human rights compliance which could then be used as another variable in studies of the factors associated with suicide in prisons at the country level.

Doing this form of analysis would require human rights lawyers to be involved in such studies in order to describe the applicable standards, and to examine whether the particular prison system complies with them or not. This second step of the process would also greatly benefit from the input of medical professionals, and those working in prisons and with prisoners to provide an assessment of human rights compliance in practice. Producing research which shows which states are complying with their human rights standards has potential benefits. First, objective, scientific examination of compliance with human rights can be used by domestic and international oversight and monitoring bodies as robust evidence on which to support their reports and recommendations. The Equality and Human Rights Commission has, for example, produced a checklist for those holding adults in detention which is based on human rights standards and is a practical tool for those seeking to fulfil their obligations (Equality and Human Rights Commission, 2015). The CPT has also created checklists for prison doctors, as noted above. Second, this evidence can also be used by domestic and international courts which do have powers of enforcement to hold prison authorities and states to account. Third, such evidence can also support civil society organisations to raise awareness and campaign on the topic. As noted below, those who stand implacably opposed to the protection of human rights may not be moved by such evidence, but without it there is limited chance of change.

More generally, the drafting and analysis of human rights law on the topic of suicide in prison would be enriched considerably through the input of scientific researchers and healthcare researchers in order to ensure those standards are meaningful to those affected by them .

## Further reflections

There are no guarantees that human rights standards automatically translate into good processes or good outcomes. Anybody familiar with the raft of reports from inspection bodies, Ombudsmen and others tasked with investigating deaths in custody need no reminding of that (Muslin and Deitch, 2010). Simply knowing what those standards are is by no means a guarantee that those who are indifferent to them will implement them or take any care about them. Much more research is necessary into why prison administrations do not comply with their obligations, reasons which may include: resources; lack of interest; lack of understanding; lack of training; or lack of enforcement powers on the part of those tasked with responding to deaths in prison.

Understanding the principles which do apply can nonetheless support the promotion of good practice in prisons. Doing so allows for oversight bodies to continue to point out where prison authorities are not meeting the required human rights standard, and for courts to hold prison authorities to account. Understanding the principles also supports training programmes for staff in the area of human rights compliance. At the very least, measuring where human rights principles are respected and where they are not can assist in examinations of the factors behind suicides in prisons, and provide evidence of the connection between indifference or inability to comply with human rights standards and the stark outcome of suicide.

## References

[CR1] Bennett J (2016). The working lives of prison managers : Global change, local culture and individual agency in the late modern prison.

[CR2] Bicknell Christine, Evans Malcolm (2017). Monitoring Prisons: The Increasingly Complex Relationship Between International and Domestic Frameworks. Europe in Prisons.

[CR3] Daems Tom, Robert Luc (2017). The Future of Europe in Prisons. Europe in Prisons.

[CR4] Equality and Human Rights Commission. (2015). *Human rights framework for adults in detention*. London: Equality and Human Rights Commission.

[CR5] Fazel S, Ramesh T, Hawton K (2017). Suicide in prisons: An international study of prevalence and contributory factors. The Lancet Psychiatry.

[CR6] Girling EVI, Armstrong S, McAra L (2006). European identity, penal sensibilities, and communities of sentiment. Perpsectives on punishment: The contours of control.

[CR7] Hutchinson T, Duncan N (2015). Defining and describing what we do: Doctrinal legal research. Deakin Law Review.

[CR8] Liebling A (1992). Suicides in prison. London.

[CR9] Livingstone S, Owen T, Macdonald A (2015). Livingstone, Owen, and MacDonald on prison law.

[CR10] McCrudden S (2006). Legal research and the social sciences. Law Quarterly Review.

[CR11] Mushlin M, Deitch M (2010). Opening up a closed world: What constitutes effective prison oversight?. Pace Law Review.

[CR12] Rodley, S. N. (2015). Bringing the standards up to standard. Retrieved from https://www.penalreform.org/blog/standards-brought-up-to-standard/

[CR13] Rogan M (2014). Prison law.

[CR14] Sanchez FC, Fearn N, Vaughn MG (2018). Risk Factors Associated With Near-Lethal Suicide Attempts During Incarceration Among Men in the Spanish Prison System. Int J Offender Ther Comp Criminol.

[CR15] Simon J (2014). Mass incarceration on trial : A remarkable court decision and the future of prisons in America.

[CR16] Snacken S (2010). Resisting punitiveness in Europe?. Theoretical Criminology.

[CR17] Snacken S, Dumortier E (2012). Resisting punitiveness in Europe? : Welfare, human rights and democracy.

[CR18] Stoliker BE (2018). Attempted Suicide: A Multilevel Examination of Inmate Characteristics and Prison Context..

[CR19] United Nations Office on Drugs and Crime (2017). Assessing compliance with the Nelson Mandela rules: A checklist for internal inspection mechanisms.

[CR20] Van Zyl Smit D, Snacken S (2009). Principles of European prison law and policy : Penology and human rights.

[CR21] Whitman JQ (2003). Harsh justice: Criminal punishment and the widening divide between America and Europe.

[CR22] European Committee for the Prevention of Torture and Inhuman or Degrading Treatment or Punishment. (1993). *3*^*rd*^*General Report on the* CPT’s activities.

[CR23] European Committee for the Prevention of Torture and Inhuman or Degrading Treatment or Punishment. (2000). *11*^*th*^*General Report on the* CPT’s activities*.*

[CR24] European Committee for the Prevention of Torture and Inhuman or Degrading Treatment or Punishment. (2011). *21*^*st*^*General Report on the* CPT’s activities.

[CR25] European Committee for the Prevention of Torture and Inhuman or Degrading Treatment or Punishment. (2014). Report to the Finnish government on the visit carried out by the European Committee for the Prevention of torture and inhuman or degrading treatment or punishment (CPT) from 22 September to 2 October 2015*.* CPT/Inf (2015) 25.

[CR26] European Committee for the Prevention of Torture and Inhuman or Degrading Treatment or Punishment. *Report to the United Nations Interim Administration Mission in Kosovo (UNMIK) on the visit to Kosovo* carried out by the European Committee for the Prevention of Torture and Inhuman or Degrading Treatment or Punishment (CPT) from 15 to 22 April 2015a.* CPT/Inf (2016) 23.

[CR27] European Committee for the Prevention of Torture and Inhuman or Degrading Treatment or Punishment. (2015b). Report to the Austrian Government on the visit to Austria carried out by The European Committee for the Prevention of torture and inhuman or degrading treatment or punishment (CPT) from 22 September to 1 October 2014*.* CPT/Inf (2015) 34.

[CR28] European Committee for the Prevention of Torture and Inhuman or Degrading Treatment or Punishment. (2016a). 26^th^ General Report on the CPT’s activities*.*

[CR29] European Committee for the Prevention of Torture and Inhuman or Degrading Treatment or Punishment. (2016b). Report to the Maltese government on the visit to Malta carried out by the European Committee for the Prevention of torture and inhuman or degrading treatment or punishment (CPT) from 3 to 10 September 2015*.* CPT/Inf (2016) 25.

[CR30] European Committee for the Prevention of Torture and Inhuman or Degrading Treatment or Punishment. (2016c). Report to the Swedish government on the visit to Sweden carried out by the European Committee for the Prevention of torture and inhuman or degrading treatment or punishment (CPT) from 18 to 28 may 2015*.* CPT/Inf (2016) 1.

[CR31] European Committee for the Prevention of Torture and Inhuman or Degrading Treatment or Punishment. (2016d). Report to the government of Serbia on the visit to Serbia carried out by the European Committee for the Prevention of torture and inhuman or degrading treatment or punishment from 26 may to 5 June 2015*.* CPT/Inf (2016) 22.

[CR32] European Committee for the Prevention of Torture and Inhuman or Degrading Treatment or Punishment (2016). Report to the Portuguese government on the visit to Portugal carried out by the European Committee for the Prevention of torture and inhuman or degrading treatment or punishment (CPT) from 27 September to 7 October 2016. CPT/Inf.

[CR33] European Committee for the Prevention of Torture and Inhuman or Degrading Treatment or Punishment. (2017a). Report to the government of the United Kingdom on the visit to the United Kingdom Sovereign Base areas on Cyprus carried out by the European Committee for the Prevention of torture and inhuman or degrading treatment or punishment (CPT) from 9 to 11 February 2017*.* CPT/Inf (2017) 37.

[CR34] European Committee for the Prevention of Torture and Inhuman or Degrading Treatment or Punishment. (2017b). Report to the government of the Principality of Liechtenstein on the visit to Liechtenstein carried out by the European Committee for the Prevention of torture and inhuman or degrading treatment or punishment (CPT) from 20 to 24 June 2016*.* CPT/Inf (2017) 21.

[CR35] European Committee for the Prevention of Torture and Inhuman or Degrading Treatment or Punishment. (2017c). Report to the Greek government on the visits to Greece carried out by the European Committee for the Prevention of torture and inhuman or degrading treatment or punishment (CPT) from 13 to 18 April and 19 to 25 July 2016*.* CPT/Inf (2017) 26.

[CR36] European Committee for the Prevention of Torture and Inhuman or Degrading Treatment or Punishment. (2017d). Report to the government of the United Kingdom on the visit to the United Kingdom carried out by the European Committee for the Prevention of torture and inhuman or degrading treatment or punishment (CPT) from 30 march to 12 April 2016. CPT/Inf (2017) 9.

[CR37] European Committee for the Prevention of Torture and Inhuman or Degrading Treatment or Punishment. (2017e). Report to the government of “the Former Yugoslav Republic of Macedonia” on the visit to “the former Yugoslav Republic of Macedonia” carried out by the European Committee for the Prevention of torture and inhuman or degrading treatment or punishment (CPT) from 6 to 9 December 2016. CPT/Inf (2017) 30.

[CR38] European Committee for the Prevention of Torture and Inhuman or Degrading Treatment or Punishment. (2018a). Report to the Bulgarian Government on the visit to Bulgaria carried out by the European Committee for the Prevention of Torture and Inhuman or Degrading Treatment or Punishment (CPT) from 25 September to 6 October 2017*.* CPT/Inf (201) 15.

[CR39] European Committee for the Prevention of Torture and Inhuman or Degrading Treatment or Punishment. (2018b). Report to the Lithuanian Government on the visit to Lithuania carried out by the European Committee for the Prevention of Torture and Inhuman or Degrading Treatment or Punishment (CPT) from 5 to 15 September 2016*.* CPT/Inf (2018) 2.

